# *In vitro* Degradation of Antimicrobials during Use of Broth Microdilution Method Can Increase the Measured Minimal Inhibitory and Minimal Bactericidal Concentrations

**DOI:** 10.3389/fmicb.2016.02051

**Published:** 2016-12-21

**Authors:** Elodie A. Lallemand, Marlène Z. Lacroix, Pierre-Louis Toutain, Séverine Boullier, Aude A. Ferran, Alain Bousquet-Melou

**Affiliations:** ^1^TOXALIM, Université de Toulouse, Institut National de la Recherche Agronomique, ENVT, INP-Purpan, UPSToulouse, France; ^2^Université de Toulouse, ENVTToulouse, France

**Keywords:** antimicrobial, stability, degradation, MIC, MBC

## Abstract

The antibacterial activity of some antimicrobials may be under-estimated during *in vitro* microbiological susceptibility tests, due to their instability under such conditions. The *in vitro* degradation of seven widely used antimicrobials (amoxicillin, cephalexin monohydrate, cefotaxime sodium salt, ciprofloxacin, erythromycin hydrate, clarithromycin, and doxycycline hyclate) and its effect on MIC and MBC determinations was studied using the broth microdilution method, considered as the gold standard for MIC determinations. *In vitro* concentrations of antimicrobials, over a 24 h incubation period in the medium tested without bacteria, decreased from 33% for ciprofloxacin to 69% for clarithromycin. For cephalexin, cefotaxime, clarithromycin, and doxycycline which were the most degraded drugs, MIC and MBC values for one strain of *E. coli* and one strain of *S. aureus* were compared using the standard method or after *ad-hoc* drug complementation aiming at maintaining constant drug concentration. Abiotic degradation during the standard method was associated with a significant increase of the MIC (2 antibiotics) and MBC (3 antibiotics). However, the observed discrepancy (less than one twofold dilution), even for the most degraded drug for which the concentration at 24 h was reduced by two thirds, suggests that this would only be clinically significant in special cases such as slow-growing bacteria.

## Introduction

The minimal inhibitory concentration (MIC) is the lowest concentration of an antimicrobial that inhibits visible growth of a bacterial culture under a defined set of experimental conditions (Clinical and Laboratory Standards Institute, 2012). One reference method for measuring MIC is broth microdilution under standardized *in vitro* conditions, using a microdilution tray with twofold drug-dilution steps, and subsequent visual evaluation of bacterial growth or non-growth after 16–20 h of culture at 35 ± 2°C (Clinical and Laboratory Standards Institute, 2012). It is assumed that the *in vitro* concentration of the tested antimicrobial remains unchanged throughout the incubation period. However, the *in vitro* stability of antimicrobials is known to be affected by physical factors such as light, composition of the medium, pH, and temperature (Paesen et al., 1994). Such instability may lead to underestimation of the antibacterial activity of the compound in Antimicrobial Susceptibility Tests (AST) (Marchbanks et al., 1987) and thus alter the clinical decision to increase the dose, add another antimicrobial agent, or even abandon therapy with the compound. It has already been shown that the storage conditions of prepared MIC trays can reduce the biological activity of antimicrobials, depending on the drug, storage duration and temperature (Barry et al., 1976; Nickolai et al., 1985; Hwang et al., 1986; White et al., 1991). Few studies have focused on the degradation of antimicrobials during the actual incubation period (Wick, 1964; Marchbanks et al., 1987) and we are not aware of any study of the subsequent effect on minimal bactericidal concentration (MBC) determinations.

The aim of this study was to see if the incubation conditions used during the microdilution test could lead to the substantial degradation of some antimicrobials and if such degradation could affect the AST results.

## Materials and methods

### Antimicrobial degradation experiment

The tested drugs were selected among antimicrobials frequently used in human and veterinary medicine, without regard to their reported stability status. The drugs and dilutions in the microplates were as follows: Cephalexin monohydrate 0.5–256 μg/mL, amoxicillin 0.25–128 μg/mL, cefotaxime sodium salt 0.125–64 μg/mL, ciprofloxacin 0.031–16 μg/mL, erythromycin hydrate and clarithromycin 0.016–8 μg/mL and doxycycline hyclate 0.008–4 μg/mL. All drugs were tested at twofold serial dilutions. The antimicrobial agents were dissolved according to the specifications of the manufacturers (Sigma Aldrich, Saint Louis, MO for amoxicillin, cefotaxime sodium salt, ciprofloxacin, doxycyline hyclate, erythromycin hydrate and clarithromycin; ACS Dobfar, Tribiano, Italy for cephalexin monohydrate). Microdilution trays were prepared extemporaneously as previously described for MIC determination (Nickolai et al., 1985), except that 100 μL of cation-adjusted, organism-free Mueller-Hinton broth (MHB) was added to prevent any drug degradation that might be caused by bacteria. The trays were placed in a dark incubator at 37°C. At each sampling time (i.e., 0, 3, 6, 10, and 24 h), 100 μL were collected from 3 adjacent wells within less than 2 min, diluted with 900 μL of H_2_O and stored at 4°C for less than 4 h before analysis.

### Antimicrobials quantitative analysis

All antimicrobials were assayed in triplicate by LC/MS with an Acquity UPLC coupled to a Xevo triple quadrupole mass spectrometer (Waters, Milford, MA, USA). Five microliters of diluted sample were injected into a Cortecs C18 column (2.1^*^50 mm; 1.6 μm, Waters) and eluted with an H_2_O, 0.1% formic acid and acetonitrile gradient (except for cephalexin which was assayed with an H_2_O, 0.1% formic acid/acétonitrile isocratic elution). The column and autosampler temperatures were set at 40 and 10°C respectively. Samples were ionized in positive electrospray ionization mode (ESI+). The capillary voltage and source temperature were set at 2.5 kV and 150°C respectively. The desolvation temperature and nitrogen flow rate were set at 650°C and 800 L/h respectively. Argon was used as the collision gas at a flow rate of 0.12 mL/min. Quantification was by multiple reactions monitoring (MRM). The MRM transitions, cone voltage and collision energies used for the different antimicrobials are reported in Table 1. The method was validated in terms of linearity, inter-day and intra-day repeatability, selectivity and sensitivity, for each molecule (ICH, 1994). The calibration curve was obtained by injecting 3 replicates of seven calibration standards prepared in MHB and ranging from 0.01 to 50 μg/mL. Three different approaches were used to determine the linearity of the calibration curve: (1) calculation of the relative concentration residuals between the nominal concentration and the concentration obtained with the model (RCR%), (2) visual inspection of the residual distribution, and (3) application of a lack of fit test to check the goodness-of-fit of the model (Almeida et al., 2002). The selectivity of the method was characterized by injecting 6 replicates of MHB blank samples and comparing the obtained signal with those obtained at the limit of quantification (LOQ). The LOQ of each antimicrobial was the lowest concentration on the calibration curve that could be quantified with a precision lower than 20% and within an accuracy range of 80–120%. Calculations of intra-day and inter-day precisions and accuracies for each antimicrobial were based on three different days (except for cephalexin: 2 days) and with six replicates of QC samples at three concentration levels (low, middle, and high) covering the range of standard curve concentrations.

**Table 1:** **Retention times, MRM transitions, and MS parameters of seven different antimicrobials**.

**Antimicrobials**	**Retention time (min)**	**Ion parent (m/z)**	**Ion daughter (m/z)**	**Cone voltage (V)**	**Collision energy (eV)**
Amoxicillin	0.81	366.0	349.1	14	8
			113.9		22
Cefotaxime	1.49	456.0	396.1	18	12
			166.9		20
Ciprofloxacin	1.49	332.1	288.1	32	18
			245.1		24
Doxycycline	2.27	445.1	428.2	24	18
			154.0		30
Erythromycin	2.94	734.4	158.1	26	30
			576.4		20
Clarithromycin	3.5	748.5	158.1	30	30
			82.9		46
Cephalexin	3.43	348.0	158.0	14	8
			174.0		14

### Effect of antimicrobial degradation on AST

The effects of antimicrobial degradation on AST were further investigated by using the standard microdilution technique (Clinical and Laboratory Standards Institute, 2012) with or without an *ad-hoc* compensatory addition of antimicrobials at 6, 12, and 18 h. Cephalexin, cefotaxime, clarithromycin, and doxycycline were selected for this purpose as they were more extensively degraded than the other drugs tested. The amount of antimicrobial degraded during each 6 h step was calculated for each antimicrobial and each well. The computed lost amounts were added in 5 μL to each 200 μL well, designated “fortified well,” at each corresponding time in an attempt to compensate for this degradation. *Escherichia coli* ATCC 25922 and *Staphylococcus aureus* HG001 strains were used as test organisms. The MBC (Marchbanks et al., 1987; Clinical and Laboratory Standards Institute, 1999) for each organism was assessed by counting the viable bacteria in the broth from all wells that inhibited visible growth at 24 h. Bacteria were counted in triplicate on tryptic soy agar supplemented with magnesium sulfate and activated charcoal to prevent antibiotic carry-over effects. Colonies were counted after overnight incubation at 37°C. The lowest concentration of the antimicrobial that killed ≥99.9% of the initial inoculum was defined as the MBC end point. MBC and MIC were determined in triplicate at 24 h and the values for the control and fortified wells were compared by Wilcoxon test, with statistical significance set at *p* ≤ 0.05.

## Results

### Analytical results

The UPLC conditions were optimized to ensure the rapid elution of all 7 antimicrobials with reliable retention times and MS ionizations to avoid storage in the autosampler. The elution was performed on a C18 column within 5 min and detection was by MRM in positive electrospray ionization (ESI+). All 7 antimicrobials gave the protonated parent [M+H]^+^ with two MRM transitions. The MRM transition with the highest intensity was selected for quantification and the second MRM transition was used for antimicrobial confirmation. These two MRM transitions, the precise time of retention, in addition to the absence of interference in the MHB blank samples corroborated the high selectivity of the method.

Calibration ranges were chosen to include the MIC most frequently reported by EUCAST for *Staphylococcus aureus* strains. They also included the reported ECOFF values for *S. aureus* strains, except for amoxicillin for which the *E. coli* ECOFF value was chosen as no data for *S. aureus* were available (EUCAST, 2016). The LOQ was established in order to be able to quantify at least 90% of antimicrobial degradation (except for doxycycline). The validation results are reported in Table 2. The accuracy for all 7 antimicrobials ranged from 92 to 111% with a precision of less than 18%. LOQ were validated at 0.01 μg/mL for cefotaxime, ciprofloxacin and the two macrolides clarithromycin and erythromycin, at 0.05 μg/mL for amoxicillin and doxycycline and at 0.1 μg/mL for cephalexin. The accuracy at the LOQ ranged from 80 to 120% with a precision lower than 18%.

**Table 2:** **Validation results for seven different antimicrobials in MHB**.

**Antimicrobials**	**Linearity (*****n*** = **3)**		**Sensitivity (*****n*** = **6)**			**Repeatability (*****n*** = **6, 3 days)**			
	**Calibration range (μg/mL)**	**Model weighting**	**LOQ (μg/mL)**	**Accuracy %**	**CV %**	**QC (μg/mL)**	**Accuracy %**	**CV intra-day %**	**CV inter-day %**
Amoxicillin	0.05–10	Linear 1/X	0.05	90	17	0.075	102	14	17
						0.25	94	9	11
						2.5	98	4	7
Cefotaxime	0.01–10	Linear 1/X^2^	0.01	115	12	0.025	96	18	16
						0.25	92	9	9
						2.5	104	5	9
Ciprofloxacin	0.01–10	Linear 1/X	0.01	120	18	0.025	102	17	15
						0.25	95	4	10
						2.5	100	7	8
Doxycycline	0.05–10	Linear 1/X	0.05	80	9	0.075	97	8	14
						0.25	97	4	7
						2.5	104	8	8
Erythromycin	0.01–10	Quadratic 1/X	0.01	111	9	0.025	111	3	7
						0.25	97	2	4
						2.5	98	8	10
Clarithromycin	0.01–10	Quadratic 1/X	0.01	116	8	0.025	106	4	18
						0.25	101	4	14
						2.5	103	10	17
Cephalexin^*^	0.1–50	Linear 1/X^2^	0.1	109	9	0.3	98	8	8
						2	100	9	10
						15	104	9	9

* *Cephalexin was assayed with a previous existing method and repeatability was evaluated on 2 days*.

### Degradation of different antibiotics during the broth microdilution test

The concentrations tested were 2, 4, and 8 μg/mL for amoxicillin (Figure S1) and cephalexin, 1, 2, and 4 μg/mL for cefotaxime, 0.25, 0.5, and 1 μg/mL for ciprofloxacin, 0.125, 0.25, and 0.5 μg/mL for erythromycin and clarithromycin and 0.0625, 0.125, and 0.25 for doxycycline. The degradation half-lives and percentages of degradation of the seven antimicrobials, measured after incubation for 24 h using the standard broth microdilution test conditions, are given in Table 3. All these drugs were unstable (degradation > 20%) at the above concentrations and conditions. Some drugs, such as clarithromycin and doxycycline hyclate, were highly degraded (more than 60%). Others, such as amoxicillin, cephalexin monohydrate, cefotaxime sodium salt and erythromycin hydrate were moderately degraded (from 40 to 60%) while ciprofloxacin showed the least degradation (less than 40%).

**Table 3:** **Half-lives and percentages of degradation after 24 h for different antimicrobials under the same conditions as in the MIC determination, apart from the absence of bacteria**.

**Antimicrobial**	Degradation half-life (h)^*^	**Percentage of degradation after 24 h (%)**
Amoxicillin	27.4	45.5
Cefotaxime	28.1	44.7
Ciprofloxacin	42.0	32.7
Doxycycline	16.9	62.6
Erythromycin	31.5	41.0
Clarithromycin	14.2	69.0
Cephalexin	20.2	56.1

* *Half-life was estimated using a monoexponential model to describe the observed decay. The means of 3 experiments are reported*.

### Consequences of antibiotic degradation on MIC and MBC determinations

The MBC and MIC values for each test organism and each drug are given in Table 4. The MIC and MBC values obtained in the fortified wells were either equal to or lower than those obtained under standard conditions (no compensation for drug lost). The AST values were significantly different for one drug out of 4 for each investigated organism. However, the differences, unrelated to the presence of microorganisms, never exceeded more than one dilution.

**Table 4:** **MIC and MBC data for 4 unstable antimicrobials against *Escherichia coli* and *Staphylococcus aureus* at 24 h with the standard microdilution method or with *ad-hoc* addition of antimicrobial every 6 h to compensate for degradation**.

**Organism tested and drug**	**Mean MIC (**μ**g/mL) [Interval]**			**Mean MBC (**μ**g/mL) [Interval]**			**No. of replicates**
	**Standard microdilution**	**With addition of drug**		**Standard microdilution**	**With addition of drug**		
***E. coli***							
Cephalexin	10.67 [8-16]	8 [8]	NS	12 [8-16]	8 [8]	NS	6
Cefotaxime	0.058 [0.031–0.063]	0.045 [0.031–0.063]	NS	0.071 [0.063–0.125]	0.049 [0.031–0.063]	*P* ≤ 0.05	7
Clarithromycin	32 [32]	32 [32]	NS	128 [128]	128 [128]	NS	4
Doxycycline	1.125 [0.5–2]	0.625 [0.5–1]	*P* ≤ 0.05	>16 [>16]	>16 [>16]	NS	8
***S. aureus***							
Cephalexin	5.5 [2–8]	4.5 [2–8]	NS	12 [8–16]	7 [4–8]	*P* ≤ 0.05	4
Cefotaxime	2 [2]	1.5 [1–2]	*P* ≤ 0.05	3.66 [2–4]	2.33 [2–4]	*P* ≤ 0.05	6
Clarithromycin	0.125 [0.125]	0.125 [0.125]	NS	0.25 [0.25]	0.25 [0.25]	NS	2
Doxycycline	0.031 [0.031]	0.031 [0.031]	NS	0.088 [0.063-0.125]	0.063 [0.063]	NS	5

*Groups significantly different (p ≤ 0.05) or not (NS)*.

## Discussion

The stability or instability of the different antimicrobials used in our study has already been reported in various liquid and solid media (Hwang et al., 1986; Marchbanks et al., 1987; Paesen et al., 1994; Erah et al., 1997). Erah et al. showed that amoxicillin and clarithromycin are stable at pH 7 in aqueous solution with a degradation half-life of respectively 153.1 h and undetectable degradation (Erah et al., 1997). However, the stability of a given drug in one set of conditions cannot be univocally extrapolated to other conditions. Indeed, in our experiment, the degradation half-lives of amoxicillin and clarithromycin in MHB at 37°C were 27.4 and 14.2 h respectively. Cefotaxime stability in solution at 24 h is reported to be 92% at 25°C and 72% at 45°C (Behin et al., 2012). The extent of degradation in MHB is greater as 44.7% of the drug was degraded at 24 h and 37°C. While it is clear that light, temperature, and pH are important factors to consider, the test medium can also influence stability of a compound (Paesen et al., 1994). Cielecka-Piontek et al. did study the effect of different injection media on stability of meropenem and clavulanate potassium in order to allow clinical administration of both compound together while maximizing stability and compatibility (Cielecka-Piontek et al., 2015). There were clear differences between media, indicating the use of one media more than others in a clinical setting even if all of them where aqueous. Differences between media can be explained by their nature (aqueous or lipophilic) or differences in the catalytic effects of degradation products(Cielecka-Piontek et al., 2015). Wick stated in 1964 that every new antibiotic should be subjected to a complete study of stability under test conditions (Wick, 1964).

Previous studies have shown that antimicrobials can be degraded during the storage of MIC trays depending on the selected drug and storage time and that a low temperature (−70°C) is usually best for storage (Barry et al., 1976; Hwang et al., 1986; Marshall et al., 2000). Problems with imipenem stability have been reported during storage of MIC trays (Nickolai et al., 1985), but it did not occured with meropenem in the same conditions (Dowzicky et al., 1994) despite also being a carbapenem, attesting the need to study each compound in a given condition. It also highlighted that every precaution should be taken to minimize the loss of moisture from the frozen trays (Barry et al., 1976). White et al. demonstrated that antimicrobial degradation during storage can also result in a decline of susceptibility as the MIC values increase with degradation and storage time (White et al., 1991). Our results confirmed that antimicrobial degradation during incubation can lead to a significant increase in the observed MIC or MBC values and hence to a bias when reporting AST results.

AST plays a major role in predicting the efficacy of an antibiotic against clinical pathogens and thus in its subsequent selection (Craig, 1998). As our study with the standard microdilution method showed that some widely-used antibiotics were degraded during incubation, we explored the possible consequences of a biased MIC estimate on AST interpretation. AST results are assigned to one of three categories (susceptible, intermediate, or resistant) based on two critical lower and upper MIC breakpoints. The susceptible and resistant classes are commonly separated by an intermediate class corresponding to just one twofold dilution (Annis and Craig, 2005). Given the narrowness of this intermediate range, such a factor of variability can seriously impact the correct classification of a pathogen if its MIC is near the breakpoints (Craig, 2000). PK/PD indices such as AUC/MIC are used to establish optimal dosing regimens and are calculated from the MIC reported for the pathogen (Craig, 1998). The impact of an incorrect MIC on the dosage regimen calculation can thus be very real.

Nevertheless, it has to be kept in mind that, in our study, the differences in MIC or MBC values due to antimicrobial degradation was never higher than one twofold dilution even if 69% degradation of clarithromycin could have suggested a higher impact on MIC and MBC values. This small effect on AST values could be related to the short bacterial generation times, which are 25 and 29 min, respectively, for our strains of *E. coli* and *S. aureus* in MHB (personal data), compared to antimicrobial degradation half-lives that are quite long in comparison to those doubling times in our study. Furthermore, a MIC value is not a simple concentration, unlike the value measured by HPLC, and actually represents the overall effect obtained over the entire incubation time. Repeated measurements of MIC using the same pathogen/drug combination commonly show a 3-fold dilution range, meaning that the standard microdilution method has an accuracy of not less than one dilution interval and that a one dilution difference can be considered as acceptable (Wexler et al., 1990; Annis and Craig, 2005).

In some specific situations requiring accurate MIC and MBC values, or when the bacterial doubling time is higher than the drug half-life, as with *Mycobacterium tuberculosis* (Srivastava et al., 2016), accurate data may be obtained by controlling the antimicrobial concentration to balance antibiotic degradation. Srivastava et al. recently described a method for measuring the MIC of ertapenem against *M. tuberculosis* (Srivastava et al., 2016). They used tubes with bacteria and different concentrations of antimicrobial and added a daily supplement to compensate for ertapenem degradation. The resulting MIC was far lower than the MIC obtained with non-compensated tubes (0.6 and mg/L respectively). Another option is to plot bactericidal time-kill curves by using an initial bacterial inoculum of 5 10^5^ UFC/mL and varying the concentrations of antimicrobial. The interaction between the antimicrobial and the bacteria can be modeled (Nielsen and Friberg, 2013) and drug degradation could be considered in the modeling process (Nielsen and Friberg, 2013). The MIC obtained with this method would correspond to the concentration of antimicrobial for which a final inoculum of 5 10^5^ UFC/mL is obtained at 20 or 24 h. Such methods are interesting for specific cases but are time-consuming and cannot be applied to standard MIC determinations. In this study, we only considered the possible action of an abiotic MHB culture medium on antimicrobial degradation, as measured by UPLC, using the strict conditions applied during microdilution testing. Antibiotic degradation during such testing can also be due to metabolism, as with serum esterases and cefotaxime, for example, (Marchbanks et al., 1987), or to the effect of microorganisms in the system (Wick, 1964). Another potential source of variation for MIC determination can be the cation concentration variability in Mueller-Hinton broth (Clinical and Laboratory Standards Institute, 1999). This problem was overcome in our study by using the same cation-adjusted Mueller-Hinton broth for all experiments.

On the basis of our results, we conclude that the microdilution method remains suitable for testing the antimicrobial susceptibility of most common pathogens, despite the concomitant degradation of some antimicrobials.

## Author contributions

EL, PT, SB, AF, and AB conceived and designed the experiments. EL and ML performed the experiments. EL, ML, AF, and AB analyzed the data. EL, ML, PT, SB, AF, and AB wrote the paper.

## Funding

The experimental assays were supported by CEVA-Sogeval (France). The funders had no role in study design, data collection or interpretation, or in the decision to submit the work for publication.

### Conflict of interest statement

The authors declare that the research was conducted in the absence of any commercial or financial relationships that could be construed as a potential conflict of interest.
