# Supplementary material for: In vitro Degradation of Antimicrobials during Use of Broth Microdilution Method Can Increase the Measured Minimal Inhibitory and Minimal Bactericidal Concentrations
Source: Front Microbiol. 2016 Dec 21;7:2051. doi: 10.3389/fmicb.2016.02051 (PMC5175475; doi:10.3389/fmicb.2016.02051)
Supplement: Supplementary file 1 [file DataSheet1.docx]

Supplementary Material

*In vitro* Degradation of Antimicrobials during Use of Broth Microdilution Method Can Increase the Measured Minimal Inhibitory and Minimal Bactericidal Concentrations

Elodie A. Lallemand^*^, Marlène Z. Lacroix, Pierre-Louis Toutain, Séverine Boullier, Aude A. Ferran and Alain Bousquet-Melou

*** Correspondence:** Elodie A. Lallemand : [e.lallemand@envt.fr](mailto:e.lallemand@envt.fr)

## Figure S1

**Supplementary Figure 1.** Amoxicillin concentrations measured by UPLC in 3 different wells of MIC trays prepared extemporaneously and stored in a dark incubator at 37°C in accordance with broth microdilution method for MIC determination. Well 5, 6 and 7 are the 8, 4 and 2 µg/mL wells, respectively.
